# Meropenem-Vaborbactam Activity against Carbapenem-Resistant *Enterobacterales* Isolates Collected in U.S. Hospitals during 2016 to 2018

**DOI:** 10.1128/AAC.01951-19

**Published:** 2020-01-27

**Authors:** Mariana Castanheira, Timothy B. Doyle, Valerie Kantro, Rodrigo E. Mendes, Dee Shortridge

**Affiliations:** a JMI Laboratories, North Liberty, Iowa, USA

**Keywords:** CRE, *Enterobacterales*, outer membrane protein

## Abstract

The activities of meropenem-vaborbactam and comparators against 152 (1.1%) carbapenem-resistant *Enterobacterales* (CRE) isolates identified among 13,929 *Enterobacterales* isolates collected from U.S. hospitals during 2016 to 2018 were evaluated. CRE rates were higher in the Middle Atlantic census division (3.5%) than in the other divisions (range, 0.0% for the West North Central division to 1.4% for the West South Central division). Among the CRE isolates, 134 carried carbapenemase genes, and these included 72 isolates carrying *bla*_KPC-3_, 51 isolates carrying *bla*_KPC-2_, 4 isolates carrying *bla*_NDM-1_, 3 isolates carrying *bla*_SME-4_, 2 isolates carrying *bla*_VIM-1_, 1 isolate carrying *bla*_OXA-232_, and 1 isolate carrying *bla*_KPC-4_. Meropenem-vaborbactam was active against 95.4% of the CRE isolates and 94.8% of the carbapenem-producing *Enterobacterales* (CPE) isolates when applying the CLSI breakpoints. All isolates producing serine carbapenemases were inhibited by meropenem-vaborbactam at ≤8 mg/liter. One Citrobacter freundii isolate carrying *bla*_KPC-3_ had a meropenem-vaborbactam MIC of 8 mg/liter and was resistant according to CLSI breakpoints (the isolate was susceptible when the EUCAST criterion of an MIC of ≤8 mg/liter for susceptible was applied), had disrupted OmpC and OmpF sequences, and overexpressed AcrAB-TolC. All carbapenemase-negative CRE isolates (*n* = 18) were inhibited by meropenem-vaborbactam at ≤4 mg/liter, and the MIC values of this combination ranged from 0.25 to 4 mg/liter. Among 7 isolates carrying metallo-β-lactamases and/or oxacillinases with carbapenemase activity, meropenem-vaborbactam susceptibility was 14.3% and 57.1% when applying CLSI and EUCAST breakpoints, respectively. CRE isolates were resistant to many comparator agents, and the most active agents were tigecycline, colistin, and amikacin (to which 63.2% to 96.7% of the isolates were susceptible). Understanding the epidemiology of CRE isolates in U.S. hospitals and the resistance mechanisms among these isolates is important to form guidelines for the treatment of infections caused by these organisms, which have high mortality rates.

## Abstract (precis)

The activities of meropenem-vaborbactam and comparators against 152 (1.1%) carbapenem-resistant *Enterobacterales* (CRE) isolates identified among 13,929 *Enterobacterales* isolates collected from U.S. hospitals during 2016 to 2018 were evaluated. CRE rates were higher in the Middle Atlantic census division (3.5%) than in the other divisions (range, 0.0% for the West North Central division to 1.4% for the West South Central division).

## INTRODUCTION

Carbapenem-resistant *Enterobacterales* (CRE) isolates have emerged worldwide, have been observed in all states within the United States, and are considered endemic in a few U.S. regions (1). Although determining the exact burden of antimicrobial resistance is challenging, the CDC attributes 600 deaths and over 9,000 infection episodes every year in the United States to CRE organisms (2). Serious infections caused by CRE organisms have a higher attributable mortality rate than those caused by isolates susceptible to carbapenems (3, 4). Patients infected with a CRE organism are less likely to receive early appropriate therapy, and this delay can be associated with the elevated mortality rates (4–6).

Until recently, the treatment of CRE infections consisted in many cases of combinations that included colistin, tigecycline, and aminoglycosides (4–6). In 2015, ceftazidime-avibactam was approved by the FDA, and this combination displayed good activity against some CRE isolates, including KPC-producing organisms (7). Despite the good *in vitro* activity of ceftazidime-avibactam (8), no randomized trials specific for CRE were performed for this combination agent, and shortly after its approval, KPC-producing *Enterobacterales* isolates resistant to ceftazidime-avibactam emerged during therapy (9–11).

Meropenem-vaborbactam was approved by the FDA in 2017 for the treatment of complicated urinary tract infections (UTIs) and pyelonephritis caused by *Enterobacterales* isolates and more recently was approved by the European Medicines Agency for the treatment of complicated UTI (including pyelonephritis), complicated intra-abdominal infections, and hospital-acquired pneumonia (including ventilator-associated pneumonia) caused by *Enterobacterales* and Pseudomonas aeruginosa isolates (12). Meropenem-vaborbactam was developed to be active against KPC-producing *Enterobacterales* isolates, and its dosing regimen was designed to cover isolates with MIC values up to 8 mg/liter (13, 14). Beyond the clinical trial that led to the approval of meropenem-vaborbactam (15), the activity of this combination agent against CRE infections was also evaluated in the TANGO II trial (16). Despite the small number of isolates in both arms, 32 patients were randomized in the meropenem-vaborbactam arm and 15 were treated with the best available therapy, which included combination regimens. The analysis of that study concluded that meropenem-vaborbactam monotherapy showed significant improvement in clinical cure rates, lower nephrotoxicity, and lower mortality rates compared to the best available therapy, which consisted of multiple agents combined and included tetracyclines, aminoglycosides, colistin, and high carbapenem doses (16). Moreover, the authors highlighted that meropenem-vaborbactam considerably improved mortality rates among immunocompromised patients, who can be at a higher risk of these infections and who are rarely addressed in clinical trial studies (16).

The *in vitro* activity of meropenem-vaborbactam has been evaluated against worldwide *Enterobacterales* isolates, including CRE, multidrug-resistant, and extensively drug-resistant isolates (17, 18), and in studies that targeted isolates from more limited collections (19, 20). In this study, we expanded that knowledge by evaluating the activity of meropenem-vaborbactam and comparator agents against 152 CRE isolates collected among 13,929 isolates from 31 U.S. hospitals distributed in all 9 census divisions from 2016 to 2018. Isolates were screened for carbapenemases and other β-lactam resistance mechanisms using whole-genome sequencing analysis and evaluation of the transcription levels of genes involved in β-lactam resistance.

## RESULTS

**FIG 1:**
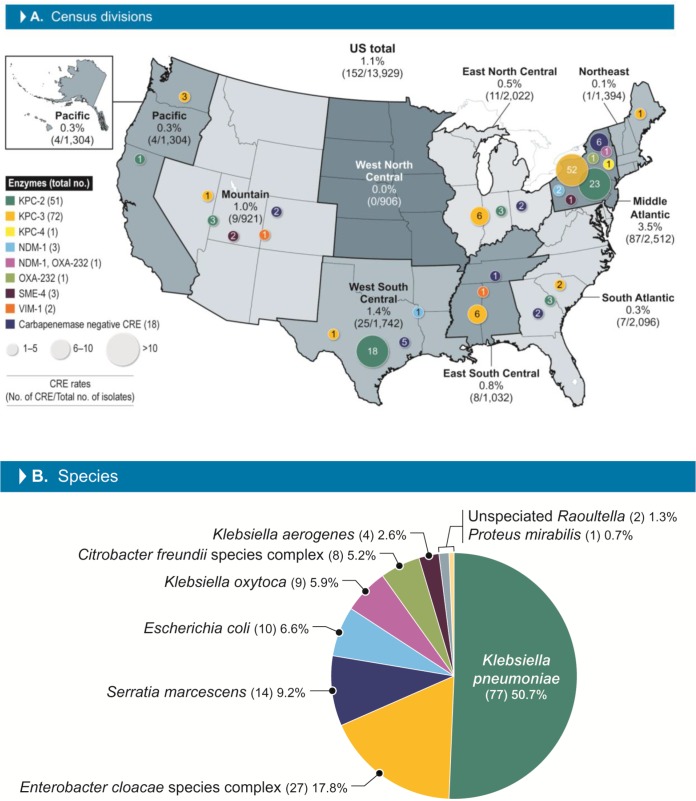
CRE distribution in U.S. census divisions (A) and species (B). CRE, carbapenem-resistant *Enterobacterales*.

A total of 152 CRE isolates (1.1% of the overall isolates) were observed among 13,929 isolates collected in 31 U.S. hospitals distributed in all 9 census divisions of the United States from 2016 to 2018. More than half of these isolates were detected in the Middle Atlantic division (*n *= 87; 57.2% of the CRE isolates; Fig. 1). This was reflected in the overall meropenem susceptibility rates for *Enterobacterales*, which were lower in the Middle Atlantic division (96.5% by use of the CLSI breakpoint; data not shown) than in the other U.S. census divisions. CRE rates were 1.4% in the West South Central division (25/1,742 isolates) and 1.0% in the Mountain division (9/921) and ranged from 0.0% in the West North Central division (0/906 isolates collected) to 0.8% in the East South Central division (8/1,032). The CRE isolates consisted of 77 Klebsiella pneumoniae isolates, 27 Enterobacter cloacae species complex isolates, 14 Serratia marcescens isolates, 10 Escherichia coli isolates, 9 Klebsiella oxytoca isolates, 8 Citrobacter freundii species complex isolates, 4 Klebsiella aerogenes isolates, 2 *Raoultella* isolates, and 1 Proteus mirabilis isolate.

**FIG 2:**
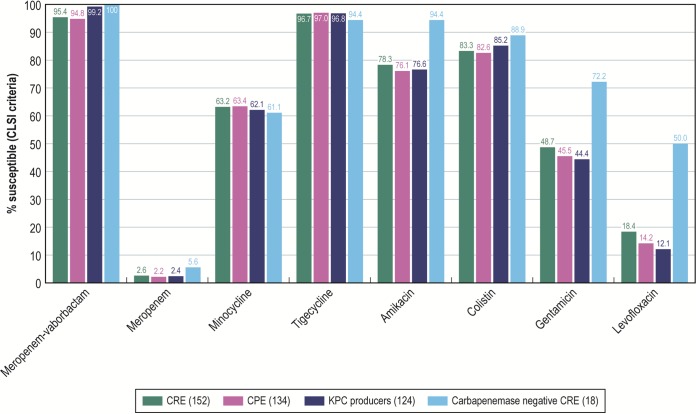
Activity of meropenem-vaborbactam and comparator agents tested against *Enterobacterales* and CRE isolates. CRE, carbapenem-resistant *Enterobacterales*; CPE, carbapenemase-producing *Enterobacterales*.

Meropenem-vaborbactam activity (MIC_50/90_, 0.06/2 mg/liter) was greater than meropenem activity (Fig. 2) and the activity of all β-lactam agents against CRE isolates. Meropenem-vaborbactam inhibited 95.4% of the CRE isolates when the CLSI breakpoint was applied and 98.0% of the isolates when the EUCAST interpretative criteria were used, whereas the other β-lactams inhibited only up to 6.6% of the CRE isolates (determined using CLSI breakpoints; data not shown). Among the other antimicrobial classes, tigecycline, colistin, amikacin, and minocycline inhibited 96.7%, 83.3%, 78.3%, and 63.2% of the CRE isolates, respectively (CLSI breakpoints were used for all antimicrobials except colistin, for which EUCAST breakpoints were applied) (Fig. 2). For all other agents, <50% of these isolates were susceptible (data not shown).

Most CRE isolates carried carbapenemase genes (134/152; 88.2%) and included 72 isolates carrying *bla*_KPC-3_, 51 isolates carrying *bla*_KPC-2_, 4 isolates carrying *bla*_NDM-1_ (1 isolate coharbored *bla*_OXA-232_), 3 isolates carrying *bla*_SME-4_ (all were S. marcescens isolates), 2 isolates carrying *bla*_VIM-1_, and 1 isolate each carrying of *bla*_OXA-232_ and *bla*_KPC-4_ (Fig. 1). Eighteen isolates were negative for carbapenemase genes. Isolates carrying *bla*_KPC-3_ were observed in all U.S. census divisions except the West North Central division, and *bla*_KPC-2_-carrying organisms were noted in all divisions except the Northeast, West North Central, and East South Central divisions. The isolates carrying *bla*_KPC-4_, the 3 isolates carrying *bla*_NDM-1_, the 1 isolate carrying *bla*_OXA-232_, and the 1 isolate carrying *bla*_SME-4_ were all detected in the Middle Atlantic division. The remaining isolate carrying *bla*_NDM-1_ was observed in the West South Central division.

Among 134 carbapenemase-producing *Enterobacterales* (CPE) isolates, meropenem-vaborbactam (MIC_50/90_, 0.03/1 mg/liter) inhibited 94.8% and 97.8% of the isolates when FDA and EUCAST breakpoints were applied, respectively (Table 1). Among the 7 isolates (<0.1% of the overall isolates and 4.6% of the CRE isolates) displaying nonsusceptible meropenem-vaborbactam MIC results when applying the CLSI breakpoint, 4 carried *bla*_NDM-1_ (and 1 of these 4 isolates also harbored an oxacillinase with carbapenemase activity [*bla*_OXA-232_]), 1 carried *bla*_OXA-232_ alone, 1 carried *bla*_VIM-1_, and 1 Citrobacter freundii isolate carried *bla*_KPC-3_. The last isolate was further investigated for additional resistance mechanisms (Table 2).

**TABLE 1:** Activity of meropenem-vaborbactam against *Enterobacterales* isolates collected in 31 U.S. hospitals during 2016 to 2018^*c*^

Organism (no. of isolates)	MIC (mg/liter)			% of isolates susceptible to meropenem-vaborbactam applying interpretative criteria of:	
	50%	90%	Range	CLSI^*a*^	EUCAST^*b*^
CRE (152)	0.06	2	≤0.015 to >32	95.4	98.0
KPC producers (124)	0.03	0.5	≤0.015 to 8	99.2	100.0
KPC-2 producers (51)	0.03	1	≤0.015 to 2	100.0	100.0
KPC-3 producers (72)	0.03	0.5	≤0.015 to 8	98.6	100.0
MBL and OXA-48-like producers (7)	8		4 to >32	14.3	57.1
CPE (134)	0.03	1	≤0.015 to >32	94.8	97.8
Carbapenemase negative (18)	1	4	0.25 to 4	100.0	100.0

a CLSI criteria are provided in reference 26.

b EUCAST criteria are provided in reference 27.

c CRE, carbapenem-resistant *Enterobacterales*; MBL, metallo-β-lactamase; CPE, carbapenemase-producing *Enterobacterales*.

**TABLE 2:**
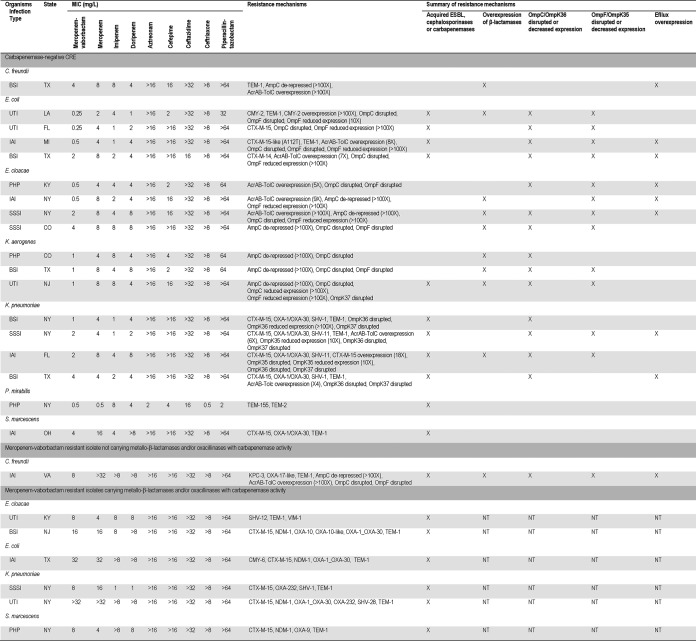
Mechanisms of resistance to β-lactams detected among carbapenemase-negative CRE isolates and isolates resistant to meropenem-vaborbactam^*a*^

a CRE, carbapenem-resistant *Enterobacterales*; BSI, bloodstream infection; UTI, urinary tract infection; IAI, intra-abdominal infection; PHP, patients hospitalized with pneumonia; SSSI, skin and skin structure infection; NT, not tested.

Meropenem-vaborbactam activity was similar for isolates carrying *bla*_KPC-2_ (51 isolates; MIC_50/90_, 0.03/1 mg/liter) and *bla*_KPC-3_ (72 isolates; MIC_50/90_, 0.03/0.5 mg/liter), and all *bla*_KPC-2_-carrying isolates and all but 1 of the *bla*_KPC-3_-carrying isolates were inhibited by meropenem-vaborbactam at ≤2 mg/liter. Isolates carrying the genes encoding KPC-4 and SME-4 displayed meropenem-vaborbactam MIC values ranging from 0.03 to 0.06 mg/liter.

One C. freundii isolate carrying *bla*_KPC-3_, in addition to *bla*_TEM-1_ and *bla*_OXA-17_-like, displayed a meropenem-vaborbactam MIC value of 8 mg/liter (intermediate by the use of CLSI breakpoints and susceptible by the use of EUCAST breakpoints). This isolate had missense mutations in OmpC and OmpF that caused the disruption of these genes and the overexpression of AcrAB-TolC and AmpC (Table 2).

Isolates negative for carbapenemase genes were also noted in all but 2 census divisions and included 4 E. cloacae species complex isolates, 4 E. coli isolates, 4 K. pneumoniae isolates, 3 K. aerogenes isolates, and 1 isolate each of the C. freundii species complex, P. mirabilis, and S. marcescens. The carbapenem resistance mechanisms among these isolates were diverse and for E. coli, E. cloacae, K. aerogenes, and K. pneumoniae included a combination of acquired β-lactamases or AmpC overexpression and disrupted sequences or reduced expression of the genes encoding OmpC/OmpK36 and/or OmpF/OmpK35. Additionally, AcrAB-TolC overexpression was observed among 2 E. coli isolates, 3 E. cloacae isolates, and 2 K. pneumoniae isolates. A noteworthy finding was that all K. pneumoniae isolates and 2 of the 4 E. coli isolates carried *bla*_CTX-M-15_-like. Among the remaining species, the C. freundii isolate had a combination of elevated expression of AmpC and AcrAB-TolC and the P. mirabilis and the S. marcescens isolates only harbored acquired β-lactamases among the mechanisms of β-lactam resistance analyzed. Meropenem-vaborbactam (MIC_50/90_, 1/4 mg/liter) inhibited all CRE isolates that did not carry carbapenemases at ≤4 mg/liter, and the MIC values for this combination ranged from 0.25 to 4 mg/liter (the MIC was 4 mg/liter for only 4 isolates). The activity of meropenem-vaborbactam was lower against these isolates than against the CRE isolates overall (MIC_50/90_, 0.06/2 mg/liter).

Meropenem-vaborbactam inhibited 1 (a 14.3% rate of susceptibility) of the 7 isolates carrying metallo-β-lactamases (MBL) and/or oxacillinases with carbapenemase activity at the current CLSI breakpoint, but 57.1% of the isolates were susceptible to this combination when using the EUCAST breakpoint criteria. One S. marcescens isolate carrying *bla*_VIM-1_ had a meropenem-vaborbactam MIC of 4 mg/liter, and 1 E. cloacae isolate harboring *bla*_VIM-1_, 1 S. marcescens isolate carrying *bla*_NDM-1_, and 1 K. pneumoniae isolate carrying *bla*_OXA-232_ had MIC values of 8 mg/liter for this combination. These isolates were resistant to meropenem alone and were susceptible to tigecycline. Minocycline and colistin inhibited 71.4% of these isolates when the CLSI and EUCAST breakpoints, respectively, were applied (data not shown).

## DISCUSSION

In a recent study by Satlin et al. (21) investigating the CRE epicenter in the United States, patients with CRE infections had a 47-h delay in appropriate therapy and 49% died within 30 days. In another U.S. investigation, mortality rates among patients with CRE infections ranged from 13.3%, when using combination therapy that included colistin, tigecycline, and aminoglycosides, to 57.8%, when patients were treated with monotherapy (22). Similar data were replicated in Greece and Italy (4, 6). These studies highlight the threat of CRE infections for patients and the importance of understanding the rates of CRE in individual institutions to establish protocols and methods to identify CRE isolates and evaluate their susceptibility patterns to implement effective therapies promptly.

While other carbapenemases have been reported in U.S. hospitals, KPC-producing organisms are still the most frequent. These isolates have been reported in high numbers in the New York City area and Texas but have been encountered in every U.S. state (https://www.cdc.gov/hai/organisms/cre/trackingcre.html). In this survey, most carbapenemase-producing isolates from 31 U.S. hospitals carried KPC-encoding genes. These isolates were observed in all U.S. census divisions, except the West North Central division. Our results confirm that meropenem-vaborbactam was very active against these isolates, and since its activity against KPC producers is remarkably greater than that of most comparator agents, the combination agent should be considered an effective alternative for the treatment of CRE infections in U.S. hospitals.

Furthermore, meropenem-vaborbactam displays activity against KPC-producing organisms regardless of the KPC variant produced; in contrast to these findings, Satlin et al. (21) observed that 42% of the K. pneumoniae isolates carrying KPC-3 had ceftazidime-avibactam MIC values of ≥4 mg/liter, while the MIC results for this combination were lower for KPC-2 producers.

Despite the elevated prevalence of isolates carrying *bla*_KPC_, isolates carrying the gene encoding NDM have been observed in 34 states, according to the CDC website (https://www.cdc.gov/hai/organisms/cre/trackingcre.html), and other carbapenemases have been sporadically detected in the United States. Meropenem-vaborbactam, like ceftazidime-avibactam and other β-lactam agents, with the exception of monobactams, is not active against isolates carrying MBLs; meropenem-vaborbactam has limited activity against isolates carrying genes encoding oxacillinases with carbapenemase activity, such as OXA-48. Noteworthy in this study was the finding that >50% of the isolates carrying MBLs and/or oxacillinases with carbapenemase activity had a meropenem-vaborbactam MIC value of ≤8 mg/liter. It would be of utmost importance to understand the efficacy of meropenem-vaborbactam for the treatment of infections caused by isolates harboring genes encoding MBLs or oxacillinases with carbapenemase activity and displaying lower MIC values against this combination. Regardless, safe and efficacious therapeutic options are still needed for the treatment of infections caused by isolates producing MBLs and some isolates producing OXA-48-like enzymes, for which treatment options are limited.

Few studies have analyzed the mechanisms of resistance among carbapenemase-negative CRE isolates, and it is assumed that overexpression of β-lactamase and impaired permeability are the main mechanisms among these isolates. We noticed that these mechanisms can be diverse, vary in different species, and might be difficult to predict. Despite the presence of a combination of these mechanisms, meropenem-vaborbactam was active against these isolates, which are more susceptible to comparator agents than carbapenemase-producing isolates but still present a challenge for the selection of appropriate antimicrobial therapy.

In clinical and *in vitro* studies (17–19, 23, 24), meropenem-vaborbactam has been shown to be more effective than combination therapy and to have greater activity than comparator agents. This new agent should be considered as a treatment option for CRE infections in the United States, where KPC-producing isolates constitute the majority of the CRE; however, diagnostic methods for determining the type of carbapenemase and/or the susceptibility to meropenem-vaborbactam or other agents active against CRE isolates must be available to confirm that the isolate is susceptible to this agent.

## MATERIALS AND METHODS

### Bacterial isolates.

A total of 13,929 *Enterobacterales* clinical isolates were consecutively collected from 2016 to 2018 in 31 U.S. hospitals as part of the SENTRY Antimicrobial Surveillance Program according to standardized protocols. Only clinically significant isolates were included in the study (1 per patient episode). Among these isolates, CRE were defined as any isolate exhibiting a doripenem, imipenem (Proteus mirabilis and members of the indole-positive tribe *Proteeae* were not included due to their intrinsically elevated MIC values), and/or meropenem MIC value of ≥4 mg/liter and were selected for further analysis. Species identification was confirmed by matrix-assisted laser desorption ionization–time of flight mass spectrometry using a Bruker Daltonics MALDI Biotyper apparatus (Billerica, MA, USA) according to the manufacturer’s instructions.

### Antimicrobial susceptibility testing.

All isolates were susceptibility tested using the CLSI reference broth microdilution method (25). CLSI (26), EUCAST (27), or FDA (28) categorical interpretations were applied. Quality control (QC) was performed using E. coli ATCC 25922 and ATCC 35218 and K. pneumoniae ATCC 700603, BAA-1705, and BAA-2814. All QC MIC results were within acceptable ranges, as published in CLSI documents (26).

### Carbapenemase screening.

CRE isolates were submitted to whole-genome sequencing using the Nextera XT library construction protocol and index kit (Illumina, San Diego, CA, USA) according to the manufacturer’s instructions and were sequenced on a MiSeq sequencer (Illumina) with a target coverage of 30 times. FASTQ format files for each sample set were assembled independently using the *de novo* assembler SPAdes (version 3.9.0) (29) with *K* values of 21, 33, 55, 77, and 99 and the careful mode on to reduce the number of mismatches, producing a FASTA format file of contiguous sequences with the best *N*_50_ value. Software designed in-house using the target assembled sequences (30) as queries for alignment against numerous resistance determinants from the NCBI Bacterial Antimicrobial Resistance Reference Gene Database (https://www.ncbi.nlm.nih.gov/bioproject/PRJNA313047) was used to search for β-lactamase genes, and potential matches were generated with the criteria of >94% identity and 40% minimum coverage length.

### Analysis of intrinsic β-lactam resistance mechanisms.

Carbapenemase-negative CRE isolates and 1 isolate that did not carry MBLs or oxacillinases with carbapenemase activity but that displayed a meropenem-vaborbactam MIC value of 8 mg/liter were further evaluated. OmpK35/OmpF and OmpK36/OmpC sequences were analyzed and compared to the reference sequences for β-lactam-susceptible isolates using whole-genome sequencing data. The transcription levels of genes encoding the intrinsic cephalosporinases, porin, efflux, and the main acquired β-lactamases (extended-spectrum β-lactamases, transferrable cephalosporinases, and carbapenemases) were measured as previously described (31, 32).
